# Atomistic Insights into Structure and Properties of ε‑Caprolactone Oligomers

**DOI:** 10.1021/acs.jpcb.5c06385

**Published:** 2026-01-27

**Authors:** Mai Ahmed, Deniz Yilmaz, Purushottam Poudel, Felix H. Schacher, Eva Perlt

**Affiliations:** † Institute of Organic Chemistry and Macromolecular Chemistry (IOMC), 9378 Friedrich Schiller University Jena, Humboldtstraße 10, 07743 Jena, Germany; ‡ Jena Center for Soft Matter (JCSM), Friedrich Schiller University Jena, Philosophenweg 7, 07743 Jena, Germany; § HIPOLE Jena (Helmholtz Institute for Polymers in Energy Applications Jena), Lessingstrasse 12-14, 07743 Jena, Germany; ∥ Otto Schott Institute of Materials Research (OSIM), Faculty of Physics and Astronomy, Friedrich Schiller University Jena, Löbdergraben 32, 07743 Jena, Germany

## Abstract

The design of functional and sustainable materials requires a detailed understanding of the material properties and degradation mechanisms. In particular, the design of fully biodegradable polymers could allow a quick and controlled decomposition of materials before they accumulate in the environment and break down to micro- and nanoplastics. An important degradation pathway proceeds via the hydrolysis of polyesters. To obtain the best performing material candidates, a multiscale-level understanding that takes into account electronic structure combined with multiple configurations at the macroscopic scale is necessary. In this contribution, we present the extension of the multiscale Quantum Cluster Equilibrium method to oligomer materials. We showcase the first application of this methodology to oligomer systems, in particular oligo­(ε-Caprolactone). The ε-Caprolactone oligomers were synthesized and characterized comprehensively by means of NMR, SEC, DSC, and TGA. Experimentally, two melting temperatures were observed, which were predicted by theoretical calculations and are in convincing agreement.

## Graphical abstract

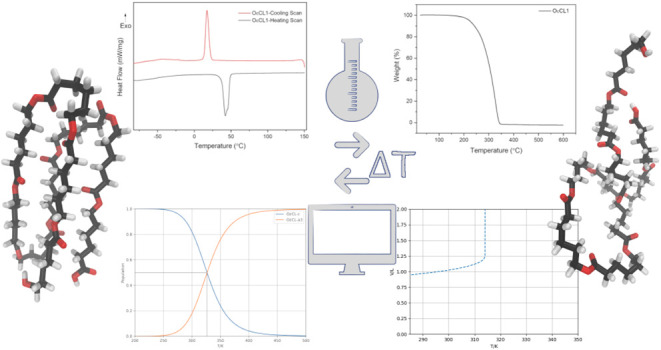

## Introduction

Polymers have been widely incorporated into modern material science, with applications ranging from packaging^1,2^ and textiles^3^ to biomedical devices,^4,5^ electronics,^6,7^ and sustainable technologies.^8^ The abundance of these stable and long-lasting materials combined with poor waste management has caused the problem of micro- and nanoplastics, which accumulate mainly in marine environment.^9^ Promising strategies to prevent the formation of micro- and nanoplastics include the prevention of the degradation into micro- and nanoparticles^9^ or the further degradation of microplastics by microorganisms.^10^ Alternatively, the design of fully biodegradable polymers would allow the implementation of proper waste treatment and reduce the amount of plastic waste that accumulates in the environment before being broken down into micro- and nanoparticles, which are impossible to eliminate.

Polyesters can undergo hydrolysis as an important step in degradation, which makes them a prospective class of compounds for biodegradable polymers. Their accessible functionalization allows one to tailor their molecular structure to achieve specific mechanical, physical, and chemical properties and furthermore improve hydrophilic properties, thereby enhancing degradability.^11–14^ Due to the ester linkages in these compounds, they are prone to hydrolytic degradation, i.e., the cleavage of ester bonds by water molecules, decreasing the polymer’s molecular weight. Functionalization by incorporation of hydroxyl (−OH) or carboxyl (−COOH) in the polymer backbone enhances hydrophilicity and water absorption, making the polymer more accessible for further break down.^15–18^ Tailoring these properties, for example of polycaprolactone, could make these polymers suitable compounds for applications such as drug delivery systems.^19^ To obtain a mechanistic understanding of these properties and processes and finally design functional degradable polymers, a theoretical model that includes quantum chemical data is essential.

Polymer systems inherently exhibit a multiscale character, and there are relevant processes happening across all scales from the atomistic monomer level up to macroscopic engineering scale. In a perspective, Schmid introduced the different theoretical techniques which address different types of polymers and, more importantly, their different properties and phenomena, which occur on different time and length scales.^20^ The most commonly used polymer theories include quantum chemical and atomistic classical simulations at the monomer and oligomer scale, generic statistical mechanics models including lattice models and off-lattice models at the polymer scale, ultracoarse-grained particle-based models at the “blob” scale of interacting polymers, field theories at the mesoscopic scale, and finally continuous field theories and transport equations at the engineering scale. However, the different time and length scales strongly overlap in polymer systems, requiring multiscale methodologies. To overcome this limitation, various flavors of static and dynamic coarse graining techniques are available. Finally, machine learning-based approaches are gaining more and more attention on different scales as well. For example, machine-learned force fields, coarse-grained potentials or the direct prediction of polymer properties based on machine-learned structure–property relationships or using other predictors were already presented, see^20^ and references therein.

Still, even with the wealth of modern theoretical tools, there remain open challenges. Two of these are strong inhomogeneities, which require a certain variety or flexibility in coarse-grained potentials and are therefore hard to cover. Similarly, defects present a type of very dilute inhomogeneity. This low concentration, or rareness, poses another challenge to the simulation, namely, the need for even larger system sizes.

Another example of a scale-bridging approach is the Quantum Cluster Equilibrium (QCE) theory,^21–23^ which has been successfully applied to a number of pure associated liquids^21,24^ as well as mixtures thereof.^25^ It has already been applied to describe the freezing of water, so it is generally applicable to solid materials.^26^ It combines a highly accurate electronic structure treatment at the cluster level with an enhanced statistical thermodynamic weighting of these clusters to obtain the macroscopic partition function and, thereby, virtually all thermodynamic potentials. Additionally, the populations of the individual clusters in combination with their quantum chemical characterization allow to compute liquid phase spectra, or even acidic constants or the ionic product of water.^27,28^ If transferred to polymer materials, this type of approach might be able to address the issues raised above: inhomogeneities may be covered by including different representative oligomer fragments. The same holds for defects, which may be introduced at the cluster level and then may be present in only small amounts, as was shown at the example of dissociated water clusters in liquid water to determine the ionic product.^27^

In this combined experimental and theoretical study, we extend the multiscale QCE theory to polymers, as they may be found in micro- and nanoplastics. We targeted oligo-(ε-Caprolactone) (OεCL) with a degree of polarization (DP) of 10 for synthesis and characterization. In parallel, a selection of decamer clusters of the OεCL was characterized using density functional theory and subsequently processed with the QCE approach. We will demonstrate the general feasibility of QCE for polymer systems and present first estimates of a phase transitions in convincing agreement with experimental observations.

## Experimental Methods

### Materials

Tin­(II) 2-ethylhexanoate (∼92.5–100%) (SnOct_2_) and benzyl alcohol (BnOH) were acquired from Sigma-Aldrich. Hydrochloric acid (HCl) (∼37%) and ε-caprolactone were obtained from ThermoFischer Scientific. Calcium hydride (CaH_2_) was purchased from J&K Scientific. All chemicals, excluding SnOct_2_ and ε-Caprolactone were used as received if not stated otherwise.

ε-Caprolactone was dried over CaH_2_ at room temperature (RT) for 24 h, distilled under reduced pressure and SnOct_2_ was dried via azeotropic distillation of toluene (3 times) before oligomer synthesis. The solvents (methanol (MeOH), tetrahydrofuran (THF) (not stabilized, HiPerSolv CHROMANORM for HPLC) and toluene) were purchased from VWR Chemicals. Toluene was dried with molecular sieves before use. The deuterated solvent, deuterated chloroform (CDCl_3_), was obtained from Deutero. PTFE syringe filters were acquired from Carl Roth under Membrane Solutions.

### Instrumentation

Proton Nuclear Magnetic Resonance (^1^H NMR) Spectroscopy measurements were performed using a 300 MHz Bruker AC spectrometer with deuterated chloroform (CDCl_3_) as the solvent. The recorded spectra were adjusted in Mestrenova, referenced to the residual solvent peak, and underwent automatic baseline and phase corrections.

Size Exclusion Chromatography (SEC) measurements were done on a Schimadzu 10er Series system with a CBM-20A controller, a DGU-14A degasser, a LC-10AD vp pump, a SIL-10AD vp autosampler, a CTO-10A vp oven, RID-10A, SPD-10AD VP, ETA- 2010, SLD 7100 detectors, and a PSS DV guard/Linear M (5 μm particle size) column. THF was utilized as the eluent with an elution rate of 1 mL min^–1^ at a temperature of 30 °C and the calibration was performed by polystyrene (PS) standards.

Differential Scanning Calorimetry (DSC) and Thermogravimetric Analysis (TGA) measurements were carried out on a NETZSCH DSC 204F1 Phoenix differential scanning calorimeter and on a NETZSCH TG 209F1 Libra thermobalance. TGA measurements were done under a nitrogen atmosphere (N_2_) in a temperature range from 25 to 600 °C with a heating rate of 20 K min^–1^. DSC measurements were performed under a nitrogen atmosphere (N_2_) (20 mL min^–1^) in a temperature range from −110 to 150 °C with heating and cooling rates of 10 K min^–1^. Second heating and cooling scans were used for evaluation.

Small angle X-ray scattering (SAXS) measurements were conducted on an Anton Paar SAXSpoint 5.0 SAXS system (Anton Paar, Graz, Austria) equipped with a Primux 100 microfocus X-ray source (Cu Kα radiation; λ = 1.54Å), ASTIX 2D multilayer X-ray optics, and a 2D EIGER2 R 1 M hybrid photon-counting detector shielded with a Mylar film (Dectris, Baden, Switzerland). The oligomer sample was put in a powder cell containing two polyimide windows and packed densely. The sample-source distance was adjusted to 1621 nm for SAXS measurements and 64.2 nm for wide-angle X-ray scattering (WAXS) measurements. The oligomer was heated up to 50 °C prior to its measurements to erase thermal history and cooled down to 25 °C at which the measurements were carried out. The data were processed with corrections for sample transmission, background scattering, and detector sensitivity. The analyses of the obtained data from SAXS and WAXS measurements were carried out in OriginPro 2022 through the peak analysis tool. The baseline of the WAXS pattern was manually fitted, and the peaks were manually selected. The determination of the degree of crystallinity (*X*_c_) involved the utilization of calculation of the areas under the peaks and under the entire curve. The determination of the crystallite sizes (*D*_hkl_) included the fitting of the most prominent diffraction peaks with a basic Gaussian function for the calculation of the full width at half-maximum.

### Syntheses of the εCL Oligomer (OεCL)

The synthesis of the oligomer of εCL with a desired degree of polymerization of 10 followed a procedure adapted from the methods given by Ren et al.^29^ and El Habnouni et al.^30^ The synthesis revolved around the subsequent addition of εCL (5 mmol, 570 mg, 554 μL), anhydrous toluene (2 mL), SnOct_2_ (0.05 mmol, 20.2 mg, 16 μL) and BnOH (0.5 mmol, 54 mg, 52 μL) to a dry Schlenk flask under constant argon flow. The flask was then sealed and stirred in an oil bath at 120 °C for 1 h. The synthesis was then terminated with an excess of 1N HCl and the oligomer was recovered through its precipitation in cold methanol and was dried under vacuum. ^1^H NMR (300 MHz, CDCl_3_, δ ppm): 7.40–7.30 ppm (BnOH, aromatic, 5H), 5.12 ppm (BnOH, ArC**H**_2_O, 2H), 4.12–4.02 ppm (−CO­(CH_2_)_4_C**H**_2_O −, 2H), 3.70–3.63 ppm (−CH_2_C**H**_2_OH, 2H), 2.40–2.20 ppm (−COC**H**_2_(CH_2_)_3_CH_2_O −, 2H), 1.76–1.20 ppm (−COCH_2_C**H**_2_C**H**_2_C**H**_2_CH_2_O −, 6H).

## Computational Details

For a computational investigation of oligo­(ε-Caprolactone) with DP = 10, the corresponding decamer units were generated and subsequently subject to a Quantum Cluster Equilibrium calculation.

Within the QCE method, a macroscopic phase is represented by a set of finite cluster structures which are assumed to be in thermodynamic equilibrium.^21–23^ Compared with routine thermochemistry calculations, it includes the following corrections and improvements. The cluster equilibrium results in a temperature-dependent distribution of different cluster motifs and therefore allows one to include different configurations, which are balanced regarding enthalpic and entropic effects. The model contains two correction parameters. The mean-field parameter *a*_mf_ scales the mean-field interaction potential, which covers the effective intercluster interaction (the intracluster interaction energy is treated by the quantum chemical calculation). Furthermore, the volume available for translation is corrected for the clusters’ volumes and scaled by the exclusion volume parameter *b*_xv_. Both parameters are optimized to reproduce reference data, which can be the density at one or more temperatures or the phase transition temperature.

To set up the cluster set, one cluster was designed to be representative of a crystalline domain by arranging the decamer chain in four parallel subunits, as shown in Figure 1 (left).

**Figure 1:**
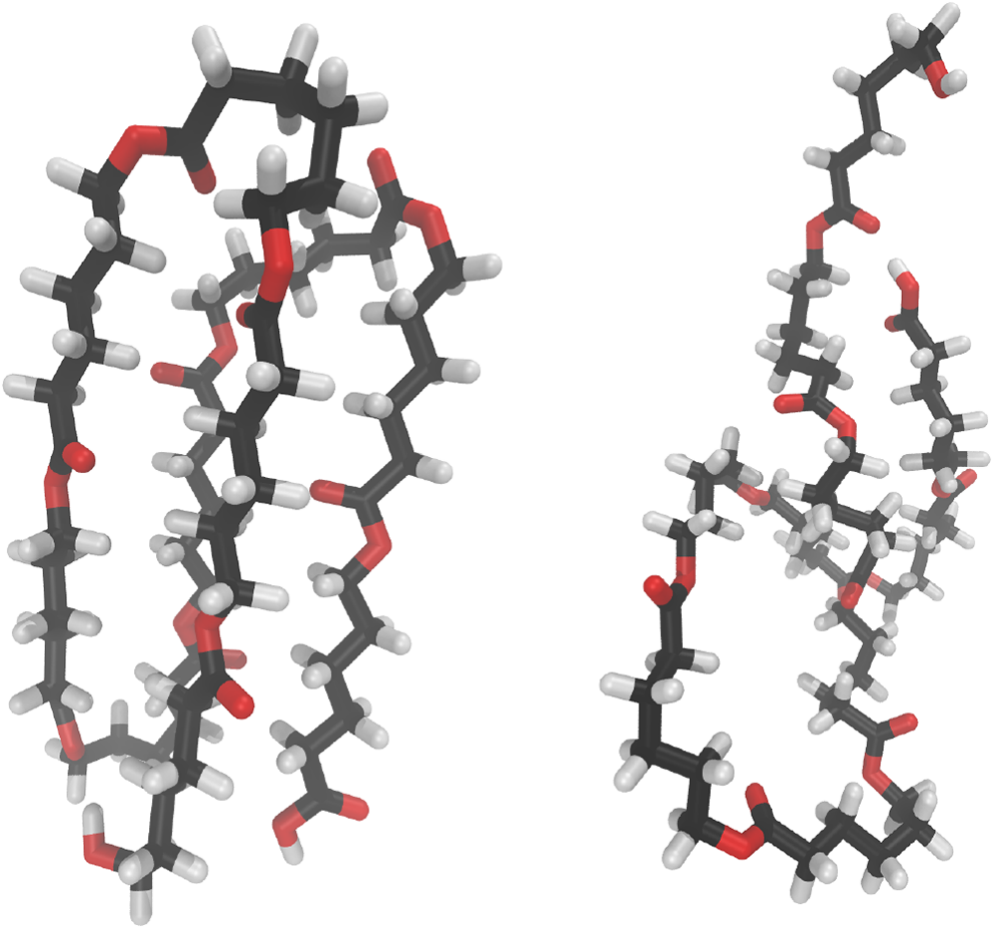
Example structures, as used in the QCE study. Crystalline motif (OεCL-c, left) as well as one exemplary amorphous structure (OεCL-a3, right).

The remaining clusters in the set represent amorphous structures in random orientations and were generated using openbabel.^31^ The conformer search tool using the genetic algorithm was applied with the default settings. The RMSD was chosen as the score property, and 25 conformers were written. All clusters were characterized computationally using the Turbomole V. 7.8.1 program suite.^32,33^ The efficient composite functional PBEh-3c was chosen.^34^ It is based on the hybrid PBE0 functional^35^ and used with the def2-mSVP basis set, the D3­(BJ) dispersion correction^36^ with Becke–Johnson damping^37^ and the inclusion of three-body terms as well as geometrical counterpoise correction. The SCF convergence criterion was chosen to be 10^–7^.

A geometry optimization was performed with a convergence criterion of 1 × 10^–4^ for the norm of the Cartesian gradient. The derivatives of quadrature weights were included in the gradient evaluations using the option weight derivatives. Harmonic vibrational analysis was performed using Turbomole’s aoforce module to confirm that the geometry is a true minimum on the potential energy surface. Furthermore, vibrational frequencies are processed in the vibrational partition function.

Apart from the crystalline structure, seven out of the 25 amorphous structures turned out to be true minimum structures, which was considered sufficient for subsequent QCE calculations. Figure 1 (right) shows one of the amorphous structures. All snapshots of the clusters are shown in Figures S1–S8 in the Supporting Information.

Subsequently, QCE calculations were performed using Peacemaker V. 4.0.0.^23^ The cluster set consisted of seven amorphous clusters, labeled OεCL-a1 to OεCL-a7, and the crystalline motif OεCL–c. Calculations were performed at standard pressure and over the temperature interval from 200 to 500 K with increments of 0.5 K. QCE calculations require electronic energies to be given as relative energies with respect to a reference cluster, which commonly is the isolated monomer in studies of associated liquids. In the present case, all clusters have the same size; therefore, the least stable structure OεCL-a6 was chosen as the reference cluster. Thus, the interaction energies were calculated as the energy difference with respect to this reference cluster in kJ mol^–1^; see Table 1.

**Table 1:** Cluster Labels and Interaction Energies in kJ mol^–1^ with Respect to the Reference Cluster OεCL-a6

cluster label	Δ*E* [kJ mol^–1^]
OεCL-c	–106.01
OεCL-a1	–18.29
OεCL-a2	–83.45
OεCL-a3	–60.23
OεCL-a4	–27.30
OεCL-a5	–67.43
OεCL-a6	0.00
OεCL-a7	–61.54

The model parameters were sampled in the range from 1.0 to 50.0 Jm^3^ mol^–2^ for *a*_mf_ and from 0.5 to 5.0 for *b*_xv_. Both intervals were chosen to be significantly larger than those for associated liquids in previous applications to allow more flexibility for this unknown system and a much larger reference species. Both parameters were optimized in four subsequent cycles, each refining the optimization interval, using the keyword grid iterations in Peacemaker. As reference data for the optimization, only the density of 1.145 g cm^–3^ at 298.15 K was chosen.

## Results and Discussion

### Oligomer Synthesis and Characterization

The synthesis of the εCL oligomer with an intended degree of polymerization (DP) of 10 was centered on a coordination–insertion ring-opening mechanism of εCL. The mentioned oligomer formation, initiated by BnOH and catalyzed by SnOct_2_, is shown in Scheme 1.

**Scheme 1:**
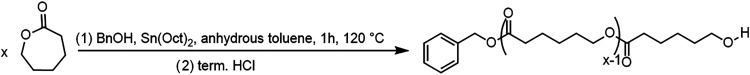
Oligomer Formation of εCL with BnOH/SnOct_2_ as the Initiator/Catalyst

The characterization of the oligomerization product of εCL with the mentioned system (OεCL1) was performed through proton NMR spectroscopy and SEC measurements. The ^1^H NMR spectrum of the acquired product is shown in Figure S9 in the Supporting Information, and its SEC chromatogram is given in the top panel of Figure 2.

**Figure 2:**
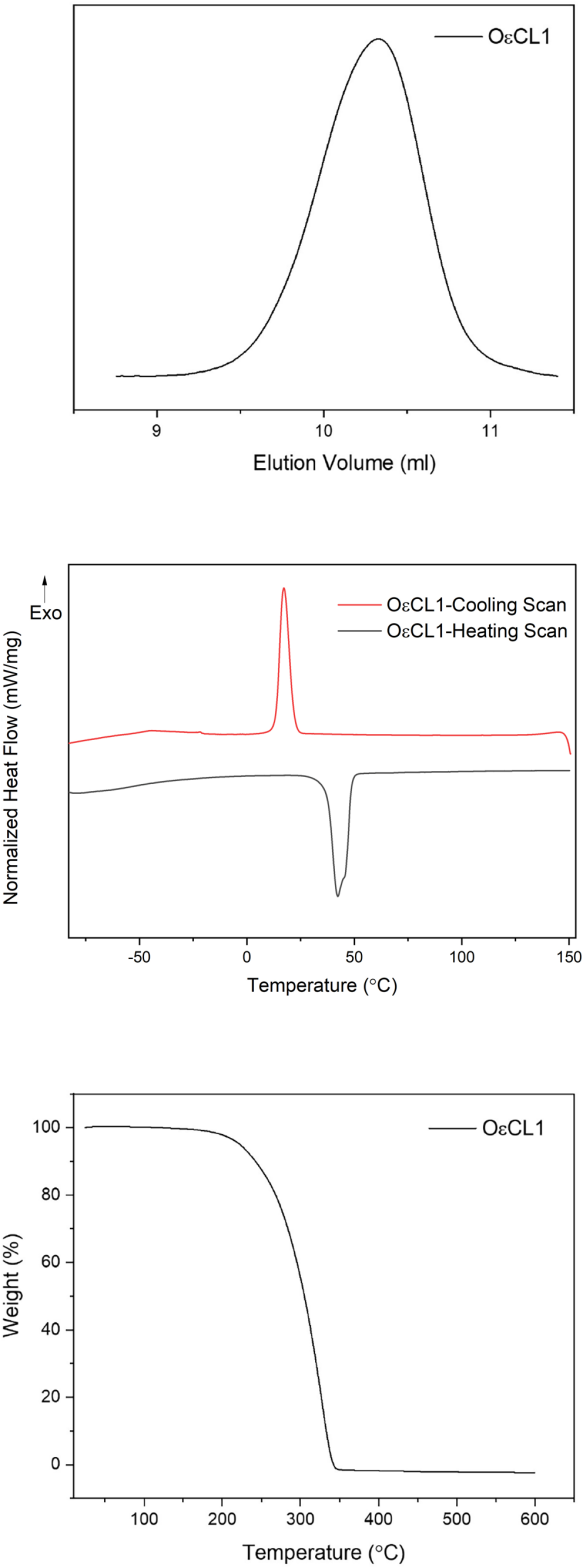
Characterization of the OεCL1 oligomer. Top, SEC chromatogram; middle, DSC heating and cooling scans; bottom, TGA curve.

The ^1^H NMR spectrum of the product confirmed the successful oligomerization of εCL, as evidenced by the desired signals of BnOH protons and those of the εCL backbone. The obtained spectrum was used to determine the DP and the $\overset{®}{M_{n}\;}$ of the oligomer. The number of εCL repeating units was acquired by comparing the integration ratios between the methylene group protons of εCL (4.12–4.02 ppm) and the aromatic protons of BnOH (7.40–7.30 ppm).

The SEC revealed a relatively narrow and monomodal molecular weight distribution. The $\overset{®}{M_{n}\;}$ value of the oligomer, calculated from the number of εCL repeating units obtained from the ^1^H NMR spectrum and its corresponding molecular weight, was also determined using SEC measurements.

The $\overset{®}{M_{n}\;}$ value obtained from the proton NMR spectrum was in accordance with the feed in terms of the degree of polymerization, whereas the one acquired from the SEC measurement was noted to be higher. The relatively low dispersity of OεCL1 and the higher $\overset{®}{M_{n}\;}$ value of OεCL1 obtained from SEC were indicative of good control over the reaction. Table 2 contains the repeating units of εCL by means of the composition of the oligomer, along with their $\overset{®}{M_{n}\;}$ values and dispersity.

**Table 2:** εCL Amount in Feed Respecting BnOH, Composition, $\overset{®}{M_{n}\;}$ Values, and Dispersity of the Oligomer

oligomer	εCL/BnOH (mmol/mmol)	composition^a^	$\overset{®}{M_{n}\;}$ ^a^,^b^ (kg mol^–1^)	$\overset{®}{M_{n}\;}$ ^c^ (kg mol^–1^)	dispersity *Đ*
OεCL1	5/0.5	O(εCL_12_)	1.5	2.7	1.24

a Determined by ^1^H NMR spectroscopy (300 MHz, CDCl_3_).

b Determined by $\overset{®}{M_{n}\;}=\left(M_{\varepsilon \mathrm{CL}}\times n_{\varepsilon \mathrm{CL}}\right)+M_{\mathrm{BnOH}}$ .

c Determined by SEC measurements (THF, PS calibration).

The εCL oligomer OεCL1, with a DP of 12, determined via ^1^H NMR spectroscopy, additionally underwent thermal and morphological characterization. The thermal characterization was performed through DSC and TGA measurements. The DSC heating and cooling scans of OεCL1 are depicted in Figure 2 (middle panel).

PεCL is a semicrystalline polyester having a melting temperature (*T*_m_) ranging from 50 to 70 °C and a glass transition temperature (*T*_g_) of around −60 °C.^38^ The aforedepicted heating scan of OεCL1 possessed split melting peaks, one at around 42 °C and the other at around 46 °C. This splitting could be attributed to the melting of the crystalline phases of PεCL, similarly observed by Ju et al.^39^ for a PεCL copolymer with pendant pyridyl disulfide groups, and to reorganizations as mentioned by Fernández-Tena et al.^38^ for PεCL with low $\overset{®}{M_{n}\;}$ values. The relatively low *T*_m_ values of OεCL1 in comparison to those of PεCL, on the other hand, were explained by the low DP and thus the low molecular weight of the oligomer. The heating scan additionally showed a *T*_g_ of around −65 °C for OεCL1, relatively similar to the one reported for PεCL.^38^

The cooling scan of OεCL1, on the other hand, displayed a crystallization temperature (*T*_c_) of around 16 °C. This value was underlined to be relatively close to the ones of PεCL samples with $\overset{®}{M_{n}\;}$ ranging from 4 kg mol^–1^ to 24 kg mol^–1^ given by Klonos et al.^40^ Therein, the *T*_c_ values of the PεCLs were between 20 and 33 °C, respectively, with consideration of the mentioned $\overset{®}{M_{n}\;}$ . It was thus plausible for OεCL1 to possess a *T*_c_ of around 16 °C, given its lower $\overset{®}{M_{n}\;}$ of 1.5 kg mol^–1^ by ^1^H NMR spectroscopy and of 2.7 kg mol^–1^ by SEC chromatography.

The acquired εCL oligomer, OεCL1, underwent TGA measurements in addition to DSC measurements, as previously mentioned. The TGA curve of OεCL1, which possesses crucial information on its stability and degradation behavior, is shown in Figure 2 (bottom panel).

The depicted TGA curve of OεCL1 is indicative of its stability up to around 205 °C. The oligomer then experienced a main degradation until around 343 °C, with almost complete weight loss. A similar stability behavior was reported by Persenaire et al.^41^ for a PεCL of $\overset{®}{M_{n}\;}$ of 1.8 kg mol^–1^, with an onset of degradation at 230 °C under helium atmosphere, extending to 600 °C. The observed degradation of OεCL1 might be attributed to a possible combination of chain cleavage by pyrolysis and unzipping depolymerization.

The morphological characterization of OεCL1 was performed through SAXS and WAXS measurements. The 1D integrated SAXS pattern of the oligomer and the corresponding WAXS data are shown in Figure 3.

**Figure 3:**
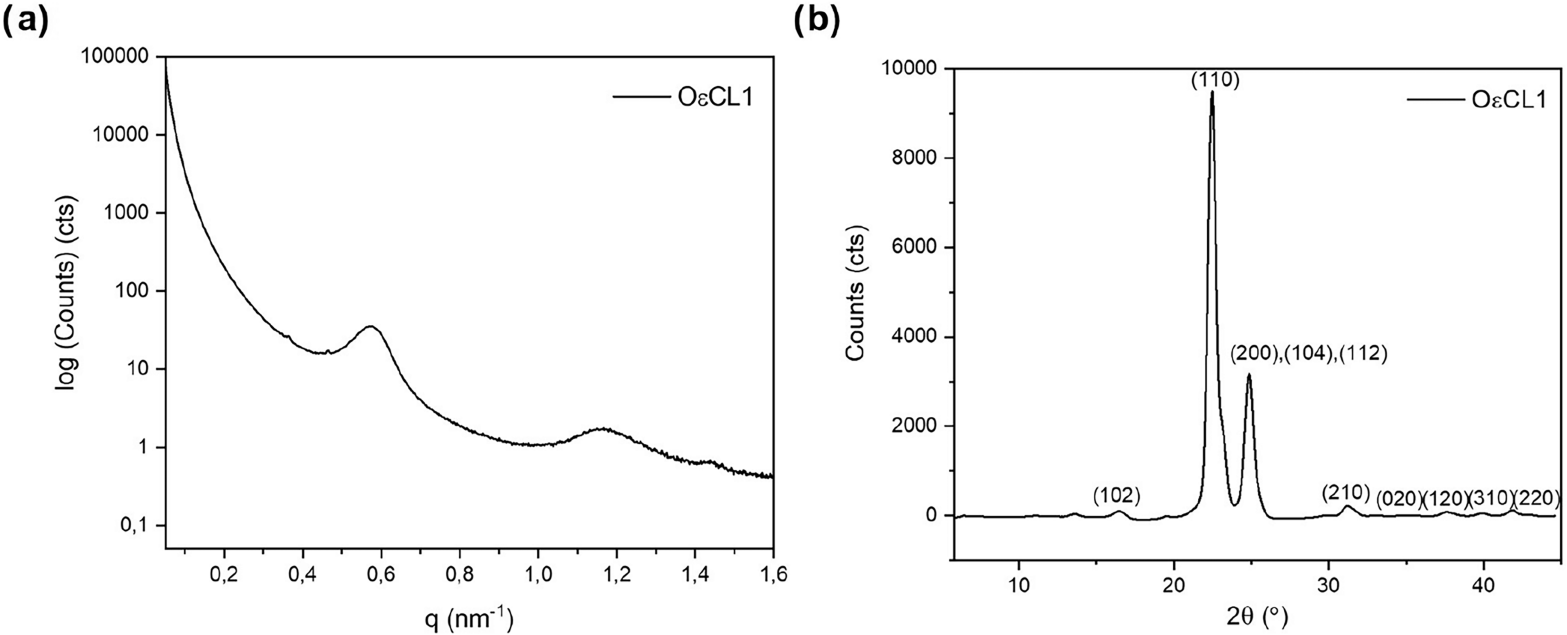
1D integrated (a) SAXS patterns of OεCL1 corresponding to *q* and (b) WAXS patterns of OεCL1 corresponding to 2*θ*.

The SAXS pattern of the oligomer shows two prominent reflexes at *q*_1_ ≈ 0.57 nm^–1^ and *q*_2_ ≈ 1.17 nm^–1^. The reflex at a *q* value between 0.4 and 0.6 nm^–1^ was attributed to long periods of crystalline lamellar structures, similarly explaining the reflex at a *q* value of 0.04 Å^–1^ in the SAXS patterns of PεCL and its Pluronics in the works of Tenorio-Alfonso et al.^42^ The near-integer ratio (*q*_2_/*q*_1_ ≈ 2) is presumably attributed to the second-order harmonics of lamellar stacking, suggesting a periodic arrangement of crystalline and amorphous layers rather than microphase separation. The first reflex corresponding to the long periods (*q*_1_) was later utilized to determine the *d*-spacing of the domains using the following equation^43^

1 $$q=\frac{2\pi }{d}$$

The morphology of OεCL1 was further investigated through WAXS measurements. The WAXS pattern of the oligomer (Figure 3 b) exhibited the expected diffraction peaks, consistent with those reported for PεCL by Nanaki et al.^44^ Here, the most prominent diffraction from (110) and (200) planes appeared at angles of 2*θ* ≈ 22.4° and 2θ ≈ 24.8°. The two crystallographic planes were utilized to determine the crystallite sizes (*D*_hkl_) in the direction perpendicular to the (hkl) plane through the Scherrer equation^45^

2 $$D_{\mathrm{hkl}}=\frac{K\lambda }{B_{\mathrm{hkl}}\cos\theta }$$

where *K* is the shape factor (typically 0.9), λ is the X-ray wavelength (0.154 nm), *B*_hkl_ is the full width at half-maximum (FWHM) of the diffraction peak in radians after instrumental correction, and *θ* is the Bragg angle.

The WAXS pattern of the oligomer was also utilized to determine its degree of crystallinity (*X*_c_) by relying on the diffractions from all of the planes through the equation given below^46^

3 $$X_{c}=\frac{A_{c}}{A_{c}+A_{a}}$$

where *A*_c_ represents the integrated area of the crystalline peaks and *A*_a_ represents the integrated area of the amorphous region in the WAXS pattern.

The *d*-spacing of the domains (*d*), the crystallite sizes (*D*_hkl_), and the degree of crystallinity (*X*_c_) of the acquired oligomer, OεCL1, are given in Table 3.

**Table 3:** Crystalline Domain Spacing or *d*-Spacing (*d*), Crystallite Sizes (*D*_hkl_), Degree of Crystallinity (*X*_c_) of OεCL1

	*D*_hkl_ (nm)		
*d* (nm)	*D*_100_	*D*_200_	X_c_ (%)
10.98	7.95 ± 0.83	7.20 ± 0.76	68.90

Crystallite sizes in the range of a few nanometers were recalled to be typical for small oligomers and poorly ordered polymers, in accordance with the values obtained for OεCL1. The aforementioned similar *D*_hkl_ values obtained for the two planes (110) and (200), on the other hand, indicated nearly isotropic crystallite dimensions. The combined results of the SAXS and WAXS measurements supported a lamellar morphology for OεCL1. The WAXS-derived crystallite sizes (7.20–7.95 nm) were smaller than the SAXS-derived long period (10.98 nm), indicating the presence of intervening amorphous layers of approximately 3–4 nm. Such lamellar structures were known to be consistent with the behavior of short-chain PεCL oligomers, which have the ability to form ordered crystalline domains separated by amorphous regions.^47^

### QCE Calculations

This first application of QCE to oligomer systems was completed successfully and smoothly, demonstrating that QCE is capable of being extended to polymer systems. The optimized parameters were determined to be *a*_mf_ = 6.230 Jm^3^ mol^–2^ and *b*_xv_ = 0.890. An excellent agreement with the experimental reference was achieved, with a calculated density of 1.145 g cm^–3^ and an error of 0.2989 × 10^–13^. It is noted that including more reference data for the density would certainly improve predictions for thermodynamic quantities, especially their temperature-dependent behavior. However, the effect of both parameters on the populations is expected to be only minor, and the one reference density chosen in this study is considered sufficient for the following analysis.

The QCE parameters are found to be somewhat different from those of associated molecular liquids, which have been extensively studied using QCE in the past. The *a*_mf_ is found to be significantly larger for the OεCL compared to values for liquids, such as water, which are typically smaller than one. The reason for this is probably the large cluster size in this study. The mean-field interaction energy depends on the density and is calculated in the following way for each cluster

4 $$E^{\mathrm{int}}=-a_{\mathrm{mf}}\frac{i}{V}$$

where *i* denotes the number of monomers in the respective cluster and *V* is the phase volume. However, in our example, the number of repeat units in each cluster is ten, and at the same time, the number of monomers *i* entered in the QCE input is one (because a smaller number of monomers generally leads to a more stable calculation). This discrepancy has no consequences for the results, and only a larger *a*_mf_ is observed to compensate for the mean-field interaction.

Also, the parameter *b*_xv_ differs from earlier studies, in which it was typically slightly larger than one. This is likely due to the close packing of crystalline motifs combined with the interpenetration of fragments of the amorphous structural motifs.

The populations of clusters with a significant population above 1% over the investigated temperature range are shown in Figure 4. Interestingly, only two clusters are populated. At lower temperatures, the crystalline cluster of OεCL-c is the only structure with a population of 1; see the blue curve in Figure 4. At ∼250 K, the population of this species decreases, while the cluster OεCL-a3, represented by the orange curve, becomes increasingly populated. Experimentally, at 25 °C, a degree of crystallinity of 68.90% was observed from the WAXS data; see Table 3. At this temperature, the QCE population of the crystalline motif is 83.44%, which is an excellent agreement, especially given the simplicity of the model, the limited cluster set, and the small number of reference data. Both curves intersect at 326.75 K (54.6 °C), and both clusters have a population of 50%, as indicated by the gray lines in Figure 4. Table 3 reports crystallite sizes of 7.95 (83) and 7.20 (76) nm. We emphasize that the cluster OεCL-c is characterized in vacuum, i.e., in the absence of periodic boundary conditions. However, constructing a hexagonal unit cell that can accommodate the structure results in cell dimensions of *a* ≈ 10Å, *b* ≈ 11.5Å, and *c* ≈ 23Å, which would indicate that crystallites may be represented as a 7 × 7 supercell of the crystalline motif.

**Figure 4:**
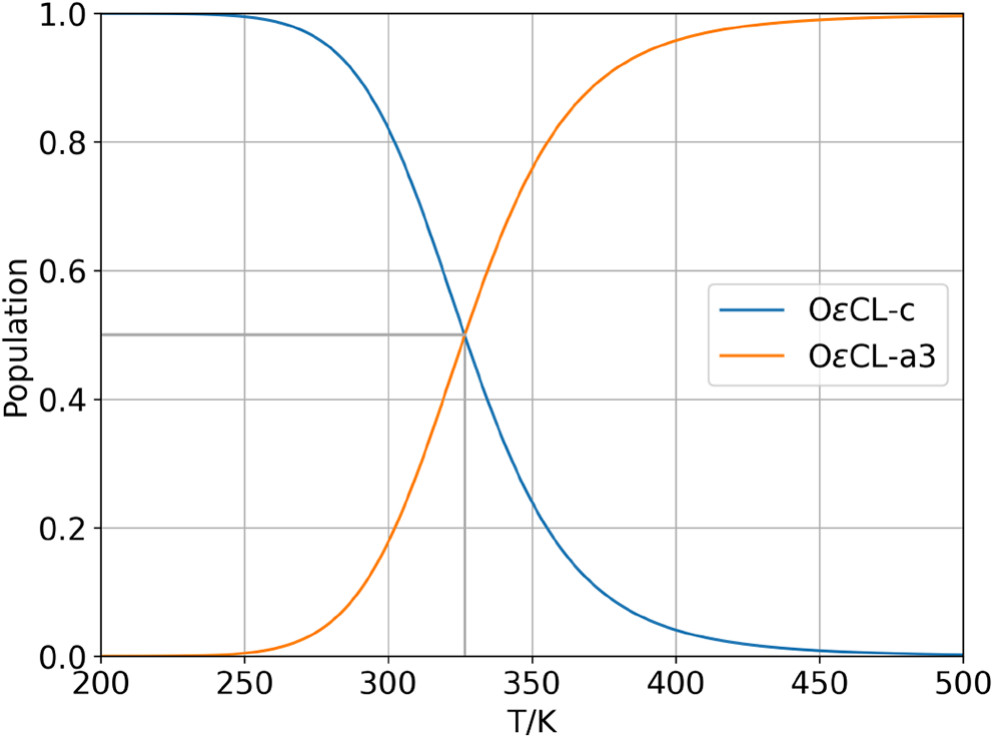
Temperature-dependent cluster populations showing only significantly populated clusters (>1%). The gray line indicates the interception of both curves.

The crystalline structure is the most stable one, as shown in Table 1, and consequently dominates the entire phase at low temperatures, in agreement with experimental observations. The great potential of the QCE approach for such systems becomes apparent at larger temperatures: by taking entropic effects into account, the enthalpic preference for the crystalline motif is counterbalanced, which leads to an increasing population of the amorphous OεCL-a3 cluster, which is less stable than OεCL-c by as much as ∼45 kJ mol^–1^. Importantly, this change in the population of cluster species is an indicator of a phase transition. However, it is interesting to note that out of seven amorphous cluster structures, a cluster that falls within the medium range of energies is exclusively populated. Even enthalpically much more favorable clusters like OεCL-a2 do not contribute at all. This again underlines the importance of vibrational effects. Furthermore, for this system consisting of rather long chains, there is likely a substantial influence of the overall shape of the cluster (coiled versus stretched), which is incorporated into the QCE method via the rotational partition function.

The calculated phase transition temperature is in astonishing agreement with the experimentally observed melting temperature (46 °C), demonstrating the general applicability of the QCE method for oligomer systems again. The deviation in the phase transition temperature of only 8 K is relatively small and can be attributed to the simplicity of the QCE method, particularly the finite cluster set.

The phase volume for 1 mole of the OεCL decamers is shown in Figure 5 for the temperature range of 285–335 K. This isobar exhibits a sharp increase in volume at 314 K (40.85 °C). This phase transition is in agreement with the experimentally observed first melting temperature. This observation is rather interesting, as the computation predicts two distinct phase transitions, which is in agreement with the two values for *T*_m_ observed in the heat flow measurements.

**Figure 5:**
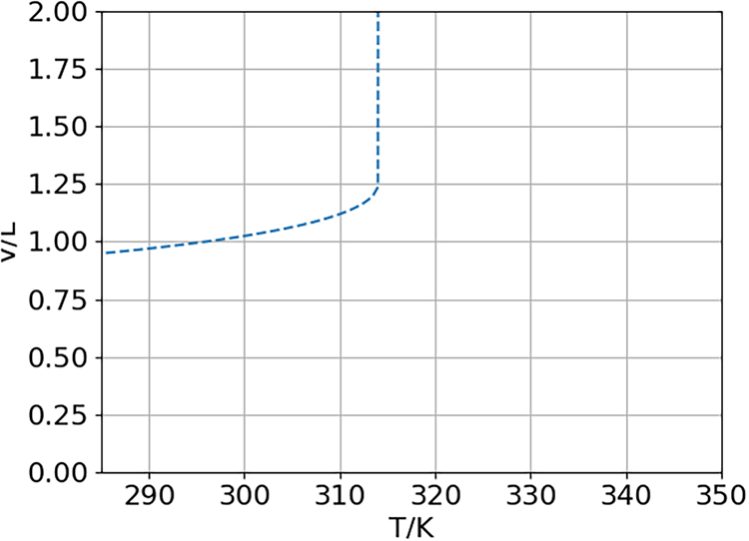
Phase volume of 1 mol OεCL. At 314 K, a sharp phase transition in the volume is observed.

The lower-temperature phase transition computationally results in a discontinuous increase in volume, while the computational parameters and the resulting cluster populations exhibit no such behavior. The reason for this can be explained by looking at how the volume is determined within QCE. It is calculated from the volume polynomial, which is a third-order polynomial derived from the van der Waals-like equation and thus can have up to three roots. Out of these, the volume that yields the lowest Gibbs free energy is chosen. In the present case, at 314 K, the volume solution that corresponds to the lower Gibbs Free Energy changes and results in an expansion of the phase. While the temperature is in perfect agreement with the experimental measurements, the magnitude of the expansion is overestimated. This, however, can be explained by the nature of the QCE theory, which is a fluid phase method, combined with the very limited cluster set and the lack of environmental effects at the cluster level.

In contrast, at the higher-temperature phase transition, the cluster configuration changes from the ordered crystalline type to the amorphous type. No further change in volume is observed here, but the change of the dominating cluster motifs clearly shows the breaking of the short-range structure.

## Conclusions

In a joint experimental and theoretical approach to well-defined OεCL decamers, we demonstrated the general applicability of the QCE method in describing oligomer systems.

The successful synthesis of the εCL oligomer with a degree of polymerization of 12 was confirmed by a combination of chromatography and spectroscopy. Thermal characterization revealed the presence of split melting peaks and a glass transition temperature similar to that of poly-(ε-caprolactone). Finally, the oligomer OεCL1 remains stable until 205 °C before it degrades in a two-step process, in agreement with previous work.

Computationally, the QCE method was successfully applied to oligomer systems for the first time. The observed population of the crystalline cluster is in surprisingly good, although not quantitative, agreement with X-ray scattering data. If adequate cluster structures are chosen, the phase volume and a transition from the crystalline phase at lower temperatures to the amorphous phase at larger temperatures can be predicted. In agreement with experimental findings, we observed two phase transitions. The first one is computationally characterized by a discontinuous change in the phase volume, while the second, at a slightly larger temperature, is observed as a change in the dominating structural motifs from crystalline to amorphous. Both temperatures agree quantitatively with the experimental melting peaks.

While these results are yet of limited value due to the limited cluster set, they demonstrate the potential of QCE when applied to oligomers and potentially also to polymer materials. For example, if more clusters for the crystalline motifs are considered and environmental effects are taken into account for all clusters, a mechanistic understanding of the nature of the phase transition can be obtained, and a more quantitative agreement with X-ray measurements can be expected. Finally, this first demonstration of the applicability of the QCE to polymer systems has important implications for future studies. By exploiting further QCE features, such as mixtures in binary QCE^25^ or the interpretation of low-populated clusters^27^ regarding low-concentration defects, further questions, for example, regarding the role of additives or the properties of copolymers, can be addressed.

## Supplementary Material



## Notes

Snapshots of all cluster structures considered in the QCE calculation (Figures S1–S8) and the ^1^H NMR spectrum of OεCL1 (Figure S9) (PDF)

## References

[1] Gupta V., Biswas D., Roy S. (2022). Materials.

[2] Tajeddin, B. ; Arabkhedri, M. Polymer Science and Innovative Applications; AlMaadeed, M. A. A. ; Ponnamma, D. ; Carignano, M. A. , Eds.; Elsevier, 2020; pp 525–543.

[3] Hu J., Chen S. (2010). J.
Mater. Chem..

[4] Moghadasi K., Mohd Isa M. S., Ariffin M. A., Mohd jamil M. Z., Raja S., Wu B., Yamani M., Bin Muhamad M. R., Yusof F., Jamaludin M. F., bin Ab Karim M. S., binti Abdul Razak B., bin Yusoff N. (2022). J. Mater. Res. Technol..

[5] Chen W.-H., Chen Q.-W., Chen Q., Cui C., Duan S., Kang Y., Liu Y., Liu Y., Muhammad W., Shao S., Tang C., Wang J., Wang L., Xiong M.-H., Yin L., Zhang K., Zhang Z., Zhen X., Feng J., Chen X. (2022). Sci. China: Chem..

[6] Ding L., Yu Z.-D., Wang X.-Y., Yao Z.-F., Lu Y., Yang C.-Y., Wang J.-Y., Pei J. (2023). Chem. Rev..

[7] Li L., Han L., Hu H., Zhang R. (2023). Mater.
Adv..

[8] Campbell I.
R., Lin M.-Y., Iyer H., Parker M., Fredricks J. L., Liao K., Jimenez A. M., Grandgeorge P., Roumeli E. (2023). Annu. Rev. Mater. Res..

[9] Jayakumar A., Radoor S., Siengchin S., Shin G. H., Kim J. T. (2023). Sci. Total Environ..

[10] Sánchez C. (2020). Biotechnol. Adv..

[11] EL-Ghoul Y., Alminderej F. M., Alsubaie F. M., Alrasheed R., Almousa N. H. (2021). Polymers.

[12] Delfi M., Ghomi M., Zarrabi A., Mohammadinejad R., Taraghdari Z. B., Ashrafizadeh M., Zare E. N., Agarwal T., Padil V. V. T., Mokhtari B., Rossi F., Perale G., Sillanpaa M., Borzacchiello A., Kumar Maiti T., Makvandi P. (2020). Prosthesis.

[13] Nashchekina Y., Chabina A., Moskalyuk O., Voronkina I., Evstigneeva P., Vaganov G., Nashchekin A., Yudin V., Mikhailova N. (2022). Polymers.

[14] Shen J., Yuan W., Badv M., Moshaverinia A., Weiss P. S. (2023). ACS Mater. Au.

[15] Leroux A., Ngoc Nguyen T., Rangel A., Cacciapuoti I., Duprez D., Castner D. G., Migonney V. (2020). Biointerphases.

[16] Feng S., Yue Y., Chen J., Zhou J., Li Y., Zhang Q. (2020). Environ. Sci.: Processes
Impacts.

[17] Barber V.
J., Borden M. A., Alty J. W., Tran L. D., Koerner H., Baldwin L. A., Alexanian E. J., Leibfarth F. A. (2023). Macromolecules.

[18] Castilla-Cortázar I., Más-Estellés J., Meseguer-Dueñas J., Escobar Ivirico J., Marí B., Vidaurre A. (2012). Polym. Degrad. Stab..

[19] Bhadran A., Shah T., Babanyinah G. K., Polara H., Taslimy S., Biewer M. C., Stefan M. C. (2023). Pharmaceutics.

[20] Schmid F. (2023). ACS Polym. Au.

[21] Kirchner B. (2005). J. Chem. Phys..

[22] Kirchner B. P. (2007). Phys. Rep..

[23] von
Domaros M., Perlt E., Ingenmey J., Marchelli G., Kirchner B. (2018). SoftwareX.

[24] Perlt E., Friedrich J., von Domaros M., Kirchner B. (2011). ChemPhysChem.

[25] Brüssel M., Perlt E., Lehmann S. B. C., von Domaros M., Kirchner B. (2011). J. Chem. Phys..

[26] Ludwig R., Weinhold F. (1999). J. Chem. Phys..

[27] Perlt E., von Domaros M., Kirchner B., Ludwig R., Weinhold F. (2017). Sci. Rep..

[28] Blasius J., Ingenmey J., Perlt E., von Domaros M., Hollóczki O., Kirchner B. (2019). Angew. Chem., Int. Ed..

[29] Ren J. M., Fu Q., Blencowe A., Qiao G. G. (2012). ACS Macro Lett..

[30] El
Habnouni S., Blanquer S., Darcos V., Coudane J. (2009). J. Polym. Sci., Part
A: Polym. Chem..

[31] O’Boyle N. M., Banck M., James C. A., Morley C., Vandermeersch T., Hutchison G. R. (2011). J. Cheminf..

[32] Balasubramani S. G., Chen G. P., Coriani S., Diedenhofen M., Frank M. S., Franzke Y. J., Furche F., Grotjahn R., Harding M. E., Hättig C., Hellweg A., Helmich-Paris B., Holzer C., Huniar U., Kaupp M., Marefat
Khah A., Karbalaei Khani S., Müller T., Mack F., Nguyen B. D., Parker S. M., Perlt E., Rappoport D., Reiter K., Roy S., Rückert M., Schmitz G., Sierka M., Tapavicza E., Tew D. P., Van Wüllen C., Voora V. K., Weigend F., Wodyński A., Yu J. M. (2020). J. Chem. Phys..

[33] Franzke Y. J., Holzer C., Andersen J. H., Begušić T., Bruder F., Coriani S., Della Sala F., Fabiano E., Fedotov D. A., Fürst S., Gillhuber S., Grotjahn R., Kaupp M., Kehry M., Krstić M., Mack F., Majumdar S., Nguyen B. D., Parker S. M., Pauly F., Pausch A., Perlt E., Phun G. S., Rajabi A., Rappoport D., Samal B., Schrader T., Sharma M., Tapavicza E., Treß R. S., Voora V., Wodyński A., Yu J. M., Zerulla B., Furche F., Hättig C., Sierka M., Tew D. P., Weigend F. (2023). J. Chem. Theory Comput..

[34] Grimme S., Brandenburg J. G., Bannwarth C., Hansen A. (2015). J. Chem. Phys..

[35] Adamo C., Barone V. (1999). J. Chem. Phys..

[36] Grimme S., Antony J., Ehrlich S., Krieg H. (2010). J. Chem. Phys..

[37] Grimme S., Ehrlich S., Goerigk L. (2011). J. Comput. Chem..

[38] Fernández-Tena A., Pérez-Camargo R. A., Coulembier O., Sangroniz L., Aranburu N., Guerrica-Echevarria G., Liu G., Wang D., Cavallo D., Müller A. J. (2023). Effect
of Molecular Weight on the Crystallization and Melt Memory of Poly­(ε-caprolactone)
(PCL). Macromolecules.

[39] Ju Y., Zhang M., Zhao H. (2017). Polym. Chem..

[40] Klonos P. A., Bikiaris N. D., Christodoulou E., Zamboulis A., Papageorgiou G. Z., Kyritsis A. (2022). Polymer.

[41] Persenaire O., Alexandre M., Degée P., Dubois P. (2001). Biomacromolecules.

[42] Tenorio-Alfonso A., Ramos E. V., Martínez I., Ambrosi M., Raudino M. (2023). Assessment
of the structures contribution (crystalline and mesophases) and mechanical
properties of polycaprolactone/pluronic blends. J. Mech. Behav. Biomed. Mater..

[43] Singh, P. S. Membrane Characterization; Hilal, N. ; Ismail, A. F. ; Matsuura, T. ; Oatley-Radcliffe, D. , Eds.; Elsevier, 2017; pp 95–111.

[44] Nanaki S. G., Papageorgiou G. Z., Bikiaris D. N. (2012). J. Therm. Anal. Calorim..

[45] Holzwarth U., Gibson N. (2011). Nat. Nanotechnol..

[46] Tencé-Girault S., Lebreton S., Bunau O., Dang P., Bargain F. (2019). Crystals.

[47] Wang Z., He Y., Müller A. (2023). J. Polymer.

