# Supplementary material for: Atomistic Insights into Structure and Properties of ε‑Caprolactone Oligomers
Source: J Phys Chem B. 2026 Jan 27;130(5):1675–83. doi: 10.1021/acs.jpcb.5c06385 (PMC12884469; doi:10.1021/acs.jpcb.5c06385)
Supplement: Supplementary file 1 [file jp5c06385_si_001.pdf]

Supporting Information: Atomistic Insights into Structure and Properties of ε-Caprolactone Oligomers

Mai Ahmed,[†,‡] Deniz Yilmaz,[†,‡] Purushottam Poudel,[¶] Felix H. Schacher,[†,‡,¶] and Eva Perlt[*,§]

†*Institute of Organic Chemistry and Macromolecular Chemistry (IOMC), Friedrich Schiller University Jena, Humboldtstraße 10, 07743 Jena, Germany*

‡*Jena Center for Soft Matter (JCSM), Friedrich Schiller University Jena, Philosophenweg 7, 07743 Jena, Germany*

¶*HIPOLE Jena (Helmholtz Institute for Polymers in Energy Applications Jena), Lessingstrasse 12-14, 07743 Jena, Germany*

§*Otto Schott Institute of Materials Research (OSIM), Faculty of Physics and Astronomy, Friedrich Schiller University Jena, Löbdergraben 32, 07743 Jena, Germany*

E-mail: eva.von.domaros@uni-jena.de

Cluster structures

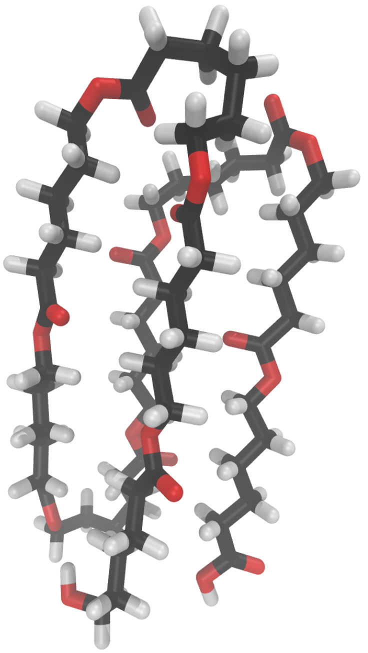

Figure S.1: Snapshot of cluster PεCL-c.

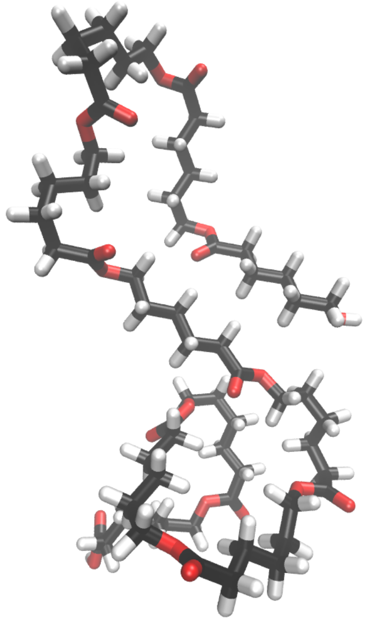

Figure S.2: Snapshot of cluster PεCL-a1.

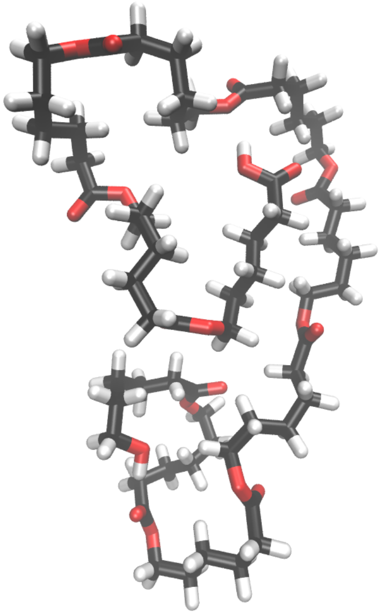

Figure S.3: Snapshot of cluster PεCL-a2.

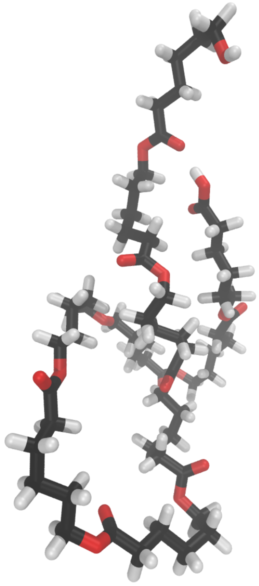

Figure S.4: Snapshot of cluster PεCL-a3.

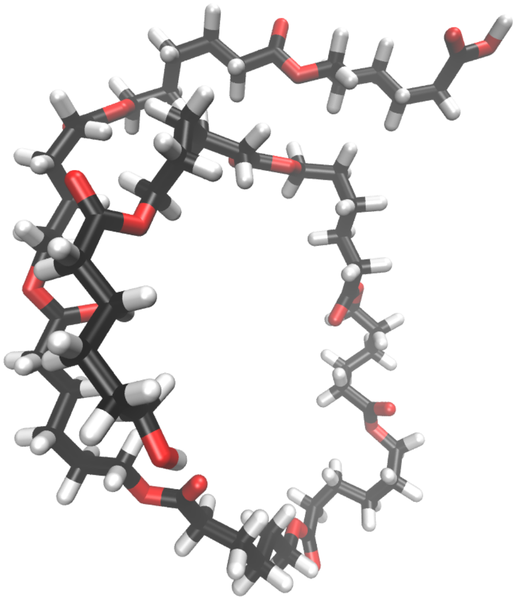

Figure S.5: Snapshot of cluster PεCL-a4.

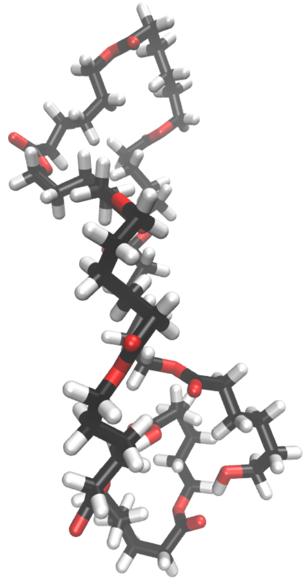

Figure S.6: Snapshot of cluster PεCL-a5.

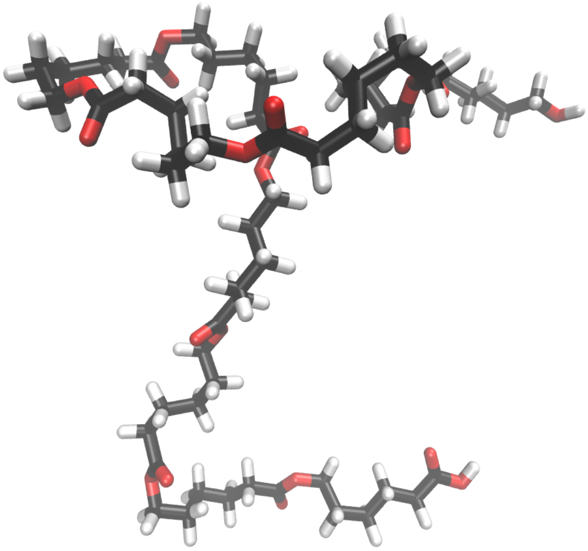

Figure S.7: Snapshot of cluster PεCL-a6.

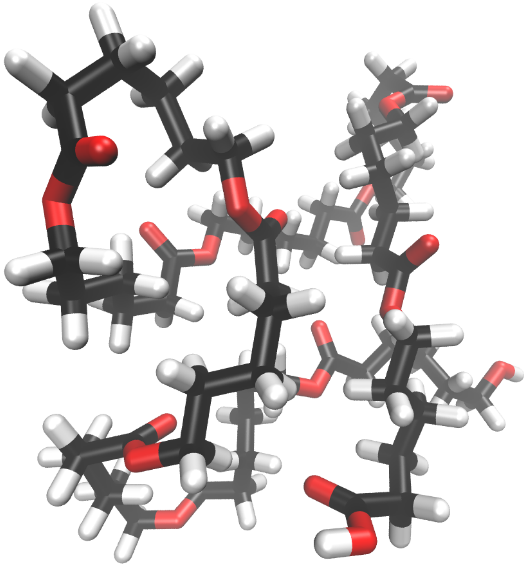

Figure S.8: Snapshot of cluster PεCL-a7.

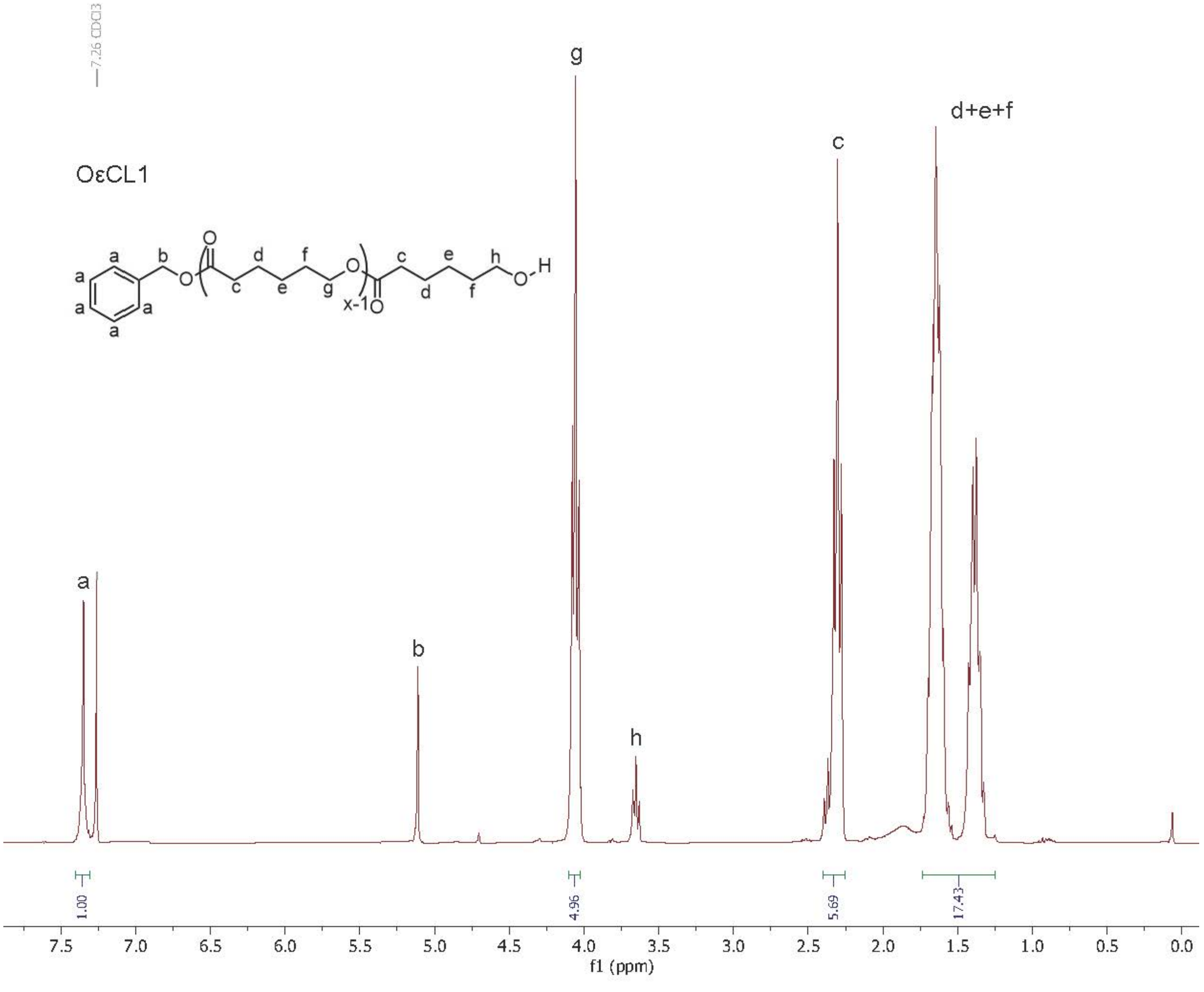

Figure S.9: ^{1}H NMR spectrum of the OεCL1 oligomer.
